# Occupational Fatalities Resulting from Falls in the Oil and Gas Extraction Industry, United States, 2005–2014

**DOI:** 10.15585/mmwr.mm6616a2

**Published:** 2017-04-28

**Authors:** Krystal L. Mason, Kyla D. Retzer, Ryan Hill, Jennifer M. Lincoln

**Affiliations:** ^1^National Institute for Occupational Safety and Health, Western States Division, CDC; ^2^Office of the Director, National Institute for Occupational Safety and Health, CDC.

During 2003–2013, fatality rates for oil and gas extraction workers decreased for all causes of death except those associated with fall events, which increased 2% annually during 2003–2013 (*1*). To better understand risk factors for these events, CDC examined fatal fall events in the oil and gas extraction industry during 2005–2014 using data from case investigations conducted by the Occupational Safety and Health Administration (OSHA). Sixty-three fatal falls were identified, accounting for 15% of all fatal events. Among fatal falls, 33 (52%) workers fell from a height of >30 feet (9 meters), and 22 (35%) fell from the derrick board, the elevated work platform located in the derrick (structure used to support machinery on a drilling rig). Fall fatalities occurred most frequently when drilling rigs were being assembled or disassembled at the well site (rigging up or rigging down) (14; 22%) or when workers were removing or inserting drill pipe into the wellbore (14; 22%). Measures that target derrickmen and workers engaged in assembling and disassembling drilling rigs (rigging up and down) could reduce falls in this industry. Companies should annually update their fall protection plans and ensure effective fall prevention programs are in place for workers at highest risk for falls, including providing trainings on proper use, fit, and inspection of personal protective equipment.

A dataset of all U.S. land-based oil and gas worker fatality investigations that occurred during 2005–2014 was provided by OSHA under a memorandum of agreement to the National Institute for Occupational Safety and Health (NIOSH). The investigations were conducted by the respective federal, state, or area OSHA office where the event occurred. The North American Industry Classification System (NAICS) was used to categorize fatal events occurring among the three types of companies in the oil and gas extraction industry: 1) oil and gas operators (companies that control and manage leased areas [NAICS 211]); 2) drilling contractors (companies that drill the wells [NAICS 213111]); and 3) well-servicing companies (companies that provide all other types of support operations that prepare a well for production and completion [NAICS 213112]) (*2*). The Occupational Injury and Illness Classification System was used to identify and code fatal fall events (*3*).

Additional variables were created by conducting key word searches of the description field of the dataset. For 15 (24%) of the 63 fatal falls, additional information was collected from media reports. A classification scheme was created that included the following variables: fall height, location of fall, activity immediately before the fall, use of fall protection, and whether the worker was securely tied to an appropriate anchor through a fall arrest or a fall restraint system.* Worker population estimates from the Bureau of Labor Statistics, Quarterly Census of Employment and Wages were used to calculate rates. Poisson regression was used to assess the trend in rates of fatal falls during 2005–2014, which were considered statistically significant if p<0.05.

Sixty-three oil and gas extraction workers died as the result of a fall during 2005–2014, (average = 6.3 fatalities per year). Fall fatality rates declined an average of 6.3% per year (incidence rate ratio = 0.937) during this time but were not statistically significant (p = 0.138). Among 61 (97%) fall-associated deaths in which the sex of the victim was known, all were male. The average age of victims was 36 years (range = 21–76 years). The majority of falls (33; 52%) were from a height of >30 feet (9 meters) (Table). Among 56 cases in which the location of the fall was known, 22 (35%) victims fell from the derrick board (Figure 1). The two most common activities occurring immediately before the fatal falls were pipe handling (14; 22%) and rigging up or rigging down (14; 22%). However, the activity occurring immediately before the fall was not determined for 22 (35%) cases.

**TABLE:** Characteristics of fatal falls among workers (N = 63) in the oil and gas extraction industry — United States, 2005–2014

Characteristic	No. (%)
**Age group (yrs)**	
20–29	23 (37)
30–39	16 (25)
40–49	10 (16)
≥50	10 (16)
Unknown	4 (6)
**Sex**	
Male	61 (97)
Female	0 (0)
Unknown	2 (3)
**Industry (NAICS code)**	
Oil and gas operators (211)	1 (2)
Drilling oil and gas wells (213111)	38 (60)
Support activities for oil and gas operations (213112)	24 (38)
**Fall height (feet)**	
0	3 (5)
1–10	4 (6)
11–20	6 (10)
21–30	6 (10)
31–40	5 (8)
41–50	5 (8)
51–60	8 (13)
61–70	3 (5)
71–80	3 (5)
≥81	9 (14)
>30	33 (52)
Unknown	11 (17)
**Location**	
Derrick board	22 (35)
Rig floor	8 (13)
Derrick ladder	5 (8)
Offshore rig	3 (5)
Stabbing board^†^	3 (5)
Other	15 (24)
Unknown	7 (11)
**Activity before fall**	
Handling/Tripping* pipe	14 (22)
Rigging up (assembling rig)	7 (11)
Rigging down (disassembling rig)	7 (11)
Maintenance	4 (6)
Welding	3 (5)
Stabbing^†^	3 (5)
Picking up sucker rods^§^	2 (3)
Picking up tools from scaffold	1 (2)
Unknown	22 (35)
**Use of fall protection equipment**	
Fall protection required	54 (86)
No fall protection required	9 (14)
Fall protection required:	
Fall protection used	24 (44)
No fall protection used	7 (13)
Unknown whether fall protection used	23 (43)
Fall protection used:	
Fall protection not anchored	15 (63)
Fall protection not worn properly	2 (8)
Equipment failure	7 (29)

**Abbreviation:** NAICS = North American Industry Classification System.

* Tripping pipe is the act of pulling the drill string out of the wellbore and then running it back in, usually to replace a worn drill bit or damaged drill pipe.

^†^ The stabbing board is a temporary platform erected on the derrick approximately 20–40 feet (6–12 meters) from the derrick floor. The crew member works on the board while cement casing is being run in a well; the worker is referred to as a “stabber” and the operation is referred to as “stabbing.” Cementing a well is the process of protecting and sealing the wellbore for the purpose of further drilling, production, or abandonment.

^§^ A series of interconnected sucker rods (steel rods, typically 25–30 feet [8–9 meters] in length, threaded at both ends) joins the visible above-ground pump jack with the pump at the bottom of the well after drilling is completed.

**FIGURE 1:**
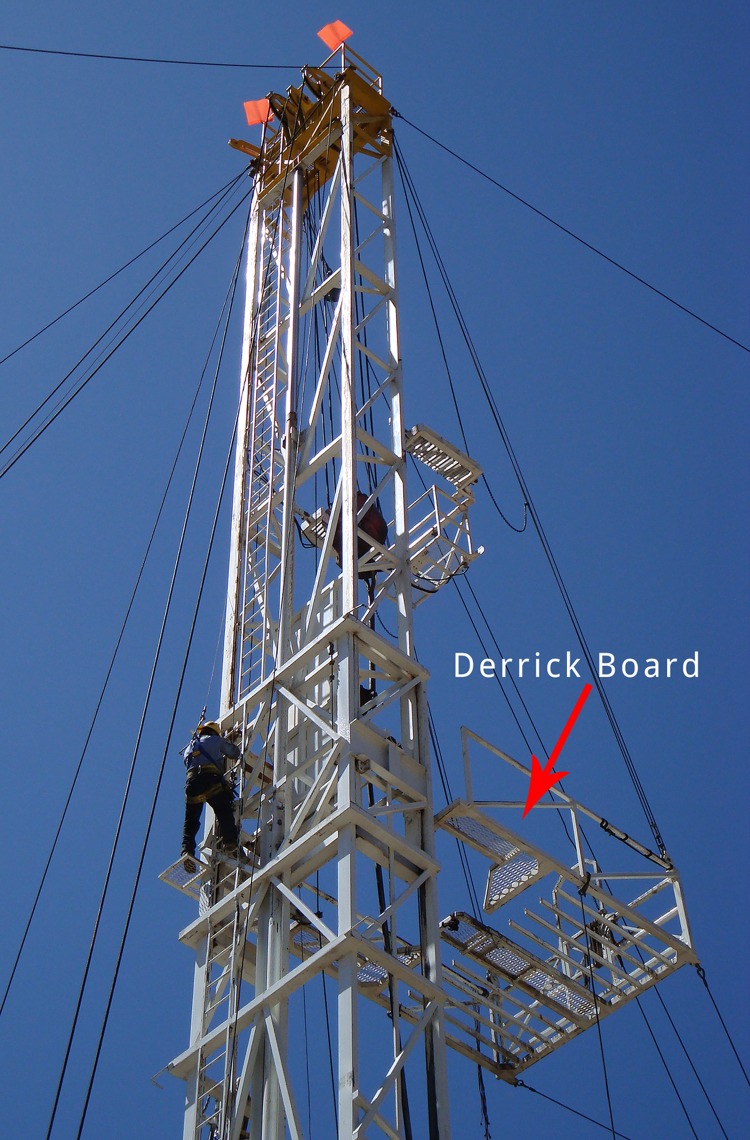
An oilfield derrickman climbing up to the derrick board

Among the three types of companies, drilling contractor workers experienced both the largest proportion of fatal fall injuries (38; 60%) and the highest fall-associated fatality rate (4.5 deaths per 100,000 workers). Twenty-four fatal fall injuries (38%) occurred among well-servicing company workers (1.1 per 100,000 workers), and only one worker death resulting from a fall occurred among oil and gas operators. Texas accounted for the largest number of fall fatalities (26; 41%), followed by Oklahoma (7; 11%) and Wyoming (6; 10%) (Figure 2).

**FIGURE 2:**
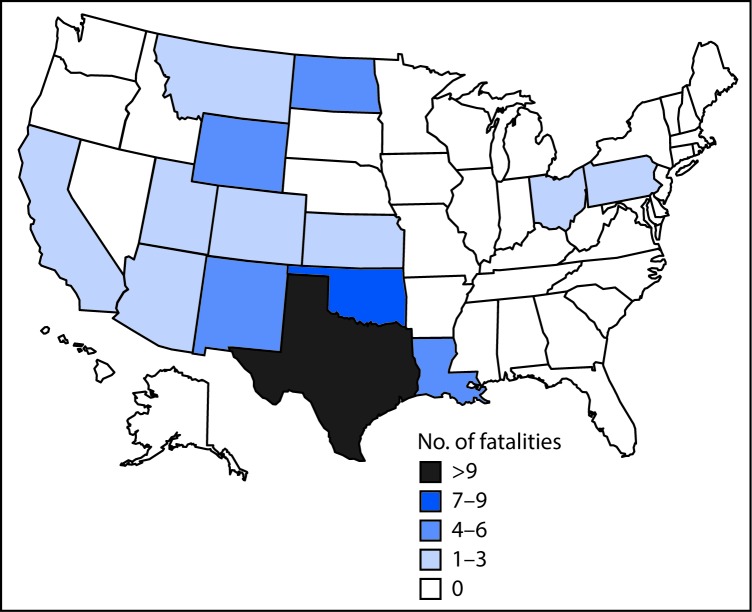
Fatalities resulting from falls in the oil and gas extraction industry (N = 63), by state — United States, 2005–2014

Fall protection equipment was required for the work being done by 54 (86%) of the 63 workers involved in fatal fall events; however, in 30 (56%) of these cases, the workers were either not using the equipment (seven) or it was not determined whether they were using the required equipment (23). Among the 24 fatally injured workers who were wearing personal fall protection equipment, 15 (63%) were not properly attached to an anchor, two (8%) were not wearing a properly fitted harness, and seven (29%) were wearing the proper harness and attached to an anchor, but the equipment failed because a retractable lifeline broke (four), a rope broke (one), the climbing assist device reportedly failed (one), or the tool ring pulled out of the harness stitching (one).

## Discussion

A 2015 analysis of occupational fatalities among oil and gas extraction workers during 2003–2013 found a decline in all leading causes of death except for fall events, which increased during that time (*1*). This analysis found slightly decreasing rates of fatal falls during 2005–2014. While the decreasing rates suggest that safety might be improving, the findings also indicate that implementation of additional interventions could prevent deaths from falls.

The majority of oil and gas extraction workers who died from a fall during 2005–2014 worked for a drilling contractor and fell from a height of >30 feet (9 meters). The occupation most commonly involved in a fatal fall were derrickmen, who work up to 90 feet (27 meters) above the rig floor on the derrick board, and handle pipe. Their work is physically demanding, repetitive, and requires a great deal of concentration. Without proper safeguards, one misstep can result in a fatal fall. Rigging up and rigging down were identified as particularly hazardous activities; one reason for this might be the opportunity for miscommunication associated with the simultaneous movements of large equipment, vehicles, and workers that occur during the these activities (*4*).

In 86% of fatal falls in this series, fall protection was required by regulation, but it was not used, was used improperly, or the equipment failed. Among the 24 fatal falls where fall protection was used, 15 (63%) workers were wearing a harness, but they fell because their harness was not attached to an anchor point. In several of these cases, a visual or verbal check between the driller and the derrickman before drilling operations began might have prevented the fall. This check would ensure that the derrickman remembers to connect his fall protection harness to both his self-retracting lifeline and a restraint system on the derrick board. Workers must also be fitted for the proper size harness and trained in proper donning of their personal fall protection equipment (*5*). Fall protection equipment should be checked daily, and equipment that is worn, heavily soiled, or damaged should be removed from service and destroyed to prevent future use. The NIOSH rig check form for harnesses and lanyards can be used to ensure inspection is thorough and that only undamaged fall protection equipment is available for use (*6*).

The findings in this report are subject to at least three limitations. First, the dataset did not contain worker fatalities that occurred outside of OSHA’s jurisdiction, such as self-employed workers, which might have resulted in an underestimate of worker fall fatalities. Second, case descriptions were the primary source of information, but they provided varying levels of detail, resulting in missing information for some variables. Finally, revisions to the Occupational Injury and Illness Classification System coding system for the event type categories occurred in 2011, and might have led to differences in the way fall fatalities were coded.

Measures in fall prevention should target derrickmen and workers engaged in rigging up and rigging down activities. Employers should first consider how to eliminate or control fall hazards using engineering controls such as automated rig technologies that allow drill pipe to be handled from the rig floor, thereby eliminating the need to work from the derrick board. Where engineering controls are not feasible, administrative controls can be implemented to ensure that derrickmen and other workers remember to anchor themselves while working at heights (*7*). Finally, training in the proper use and fit of personal protective equipment can protect workers from falls (*5*). A fall protection plan containing these processes should be available and understandable to workers, and able to be repeated by workers. The use of existing training tools and ongoing job safety analysis should be completed and shared across companies to improve hazard identification and control during rigging up and rigging down activities (*8*). In addition, training for self-rescue and rescue of fellow workers who have fallen and are suspended in the air by fall protection equipment should be written into the workplace hazard control program along with emergency response planning (*9*,*10*). Companies should ensure that plans are implemented on work sites. The oil and gas extraction industry has experienced a decline in the overall rate of fatalities. However, eliminating the need to work at height, training on how to identify and reduce the hazards of working at height, and proper use, fit, and inspections of personal protective equipment are essential in reducing fatal falls in this industry.

**Summary**

**What is already known about this topic?**

Fall events are one of the leading causes of death in the U.S. oil and gas extraction industry. During 2003–2013, fatality rates for all causes of death among oil and gas extraction workers decreased, except for deaths from falls, which increased. Workers in this industry spend a substantial amount of time working at heights, especially on the rig floor, which is located up to 30 feet (9 meters) above the ground, and in other elevated locations.

**What is added by this report?**

This is the first report to identify which categories of workers are at highest risk for fall fatalities in the U.S. oil and gas extraction industry. During 2005–2014, a total of 63 workers died in fall events. The majority of fatal fall events occurred among derrickmen who were handling pipe from the derrick board, assembling the drilling rig at the well site, or dismantling the drilling rig in preparation for transport.

**What are the implications for public health practice?**

Further research is needed to develop effective and appropriate strategies for preventing fall fatalities in the U.S. oil and gas extraction industry. Potential interventions include adopting rig technologies that eliminate the need to work at height, providing training on how to identify and reduce hazards of working at height, and ensuring proper use, fit, and inspection of personal protective equipment.

## Footnotes

* Two common types of fall protection equipment include the fall arrest system and a fall restraint system. The fall arrest system consists of a vertical lifeline, connectors, lanyard, and harness with an anchorage point overhead, serving to arrest the fall of a worker. The fall restraint system consists of a harness, lifeline and/or lanyard, and a 5,000 pound capacity anchor which keeps the worker from reaching a fall point. There are also climbing assist devices that can be used while the worker is climbing the derrick ladder.
